# Versatile modulators for laser-based FEL seeding at SwissFEL

**DOI:** 10.1107/S1600577522012073

**Published:** 2023-02-01

**Authors:** Marco Calvi, Xiaoyang Liang, Eugenio Ferrari, Arturo Alarcon, Eduard Prat, Sven Reiche, Thomas Schmidt, Didier Voulot, Kai Zhang, Romain Ganter

**Affiliations:** a Photon Science Division, Paul Scherrer Institute, 5232 Villigen, Switzerland; b Institute of Biomedical Engineering, ETH Zürich, 8092 Zürich, Switzerland; c Large Research Facility, Paul Scherrer Institute, 5232 Villigen, Switzerland; d M Division, Deutsches Elektronen-Synchrotron, Notkestraße 85, 22607 Hamburg, Germany; RIKEN SPring-8 Center, Japan

**Keywords:** soft X-ray, FEL, EEHG, insertion devices

## Abstract

The Paul Scherrer Institute is implementing laser-based seeding in the soft X-ray beamline (Athos) of its free-electron laser, SwissFEL, to enhance the temporal and spectral properties of the delivered photon pulses. This technique requires, among other components, two identical modulators for coupling the electron beam with an external laser with a wavelength range between 260 and 1600 nm. The design, magnetic measurements results, alignment, operation and also details of the novel and exotic magnetic configuration of the prototype are described.

## Abstract 2

Two modulators have been designed, assembled, magnetically tested and installed at SwissFEL in order to implement laser-based seeding at the soft X-ray beamline Athos.

## 1. Introduction

X-ray free-electron lasers (FELs) are revolutionary research instruments that allow the study of matter on spatial and time scales of atomic processes (McNeil & Thompson, 2010 ▸; Pellegrini *et al.*, 2016 ▸; Bostedt *et al.*, 2016 ▸). Most X-ray FEL facilities are based on the self-amplified spontaneous emission (SASE) process (Kondratenko & Saldin, 1980 ▸; Bonifacio *et al.*, 1984 ▸), which starts from the electrons’ shot noise and offers limited temporal coherence. A way to improve the longitudinal coherence of the FEL radiation is to start the FEL process with a coherent seed signal. This can be achieved with the self-seeding mechanism (Feldhaus *et al.*, 1997 ▸), where the seed signal is monochromated SASE radiation produced in a first stage. A better option is to use an external laser for the seed signal. To reach shorter FEL wavelengths approaching the X-ray regime, laser-based advanced schemes employing magnetic chicanes and modulators like high-gain harmonic generation (Yu, 1991 ▸) and echo-enabled harmonic generation (EEHG) (Stupakov, 2009 ▸; Rebernik Ribič *et al.*, 2019 ▸) are required. Laser-based seeding is preferred since it offers higher stability and synchronization to an external source.

SwissFEL is the X-ray FEL facility at PSI in Villigen, Switzerland. Its hard X-ray beamline, Aramis, has been under regular user operation since 2019 (Prat *et al.*, 2020 ▸). The soft X-ray beamline of SwissFEL, Athos, started user operation in 2022 ▸ (Abela *et al.*, 2019 ▸). Athos has an innovative design with flexible undulator modules (Liang *et al.*, 2021 ▸) and compact integrated chicanes (CHICs) (Prat *et al.*, 2016 ▸), offering an unprecedented control over several FEL properties such as the polarization and the peak power. The modulators presented in this article are used in a two-stage research program Hidden Entangled and Resonating Order (HERO) (Aeppli *et al.*, 2020 ▸) to manipulate the driving electron beam with an external laser signal for a better control of the temporal and spectral characteristics of the Athos FEL pulse. The first stage consists of one optical laser, one modulator and a magnetic chicane. This can be used to produce trains of attosecond pulses locked to the external laser following the ESASE scheme (Zholents, 2005 ▸; Duris *et al.*, 2021 ▸) or from energy modulation and taper (Saldin *et al.*, 2006 ▸). The installation is completed by adding a second, larger chicane and a second identical modulator to enable the operation of EEHG to obtain full control on the coherence properties of the FEL pulse. The design of the modulator supports the exploration of new operation modes beyond ESASE/energy modulation and standard EEHG. Combined with a very short laser pulse, a strong taper in the modulator field gives an ESASE current modulation with gradually growing time separation between the current spikes, supporting superradiant operation with the fresh slice technique (Tanaka *et al.*, 2016 ▸) down to even single-cycle operation of the soft X-ray beamline Athos (Tanaka, 2015 ▸). Also, by tuning half the modulator to the primary external laser wavelength and the other half to its second harmonic, the energy modulation can be tailored for a more efficient ESASE, HGHG or EEHG process (Hemsing & Xiang, 2013 ▸). Finally, the sub-period configuration enhances the performance of self-modulation for a single attosecond pulse (MacArthur *et al.*, 2019 ▸).

## 2. Design

To satisfy the above requirements, the modulators were designed with great operational flexibility, where the strength of each pole can be tuned individually, making it unique also compared with the work of Tanaka *et al.* (2021 ▸) where exclusively the strength of each period can be adjusted independently. The choice of the magnetic field amplitude was based on the period length, λ_u_ = 0.2 m, imposed by the mechanical frame of the existing CHICs and the resonance condition of the modulator, 

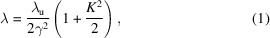

where γ is the Lorentz factor, *K* the deflection parameter defined as 

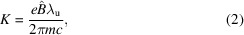


**Figure 1:**
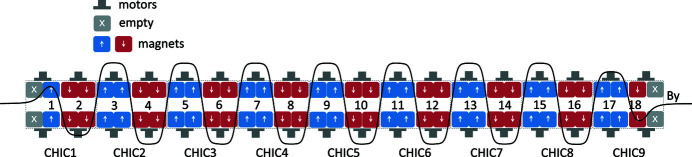
Functional view of the modulator with its nine CHIC modules with 18 independently movable magnetic poles. The black solid line is an example of the measured magnetic field profile, *By*.

**Figure 2:**
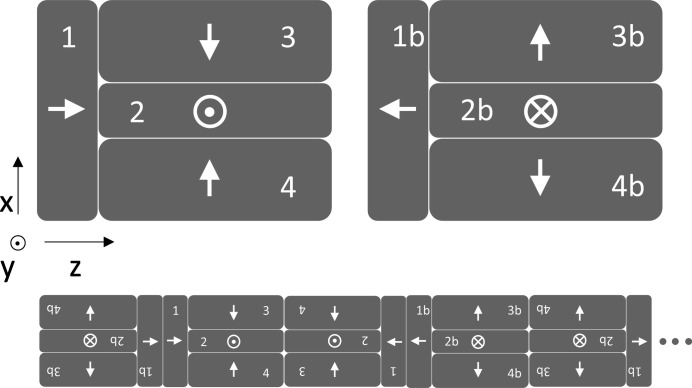
Details of the half pole magnet assembly in its *xz*-plane projection. The two polarities are presented on the left and on the right with which is possible to build the full structure.

*m* and *e* the mass and the charge of the electron and 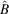 the equivalent magnetic field amplitude which shall be evaluated using the Fourier series as presented by Calvi *et al.* (2018 ▸). This is especially important for this modulator where the field profile, *B*_*y*_(*z*), is far from sinusoidal and thus the maximum amplitude does not represent well the resonance condition. Given the design electron beam energy interval between 2.75 and 3.25 GeV^1^, *K* shall range between 12 and 36 to cover the wavelength scale between 260 and 1600 nm. Each of the two modulators consists of 16 full poles and two half poles, one in each of the two extremes as shown in Fig. 1 ▸. Its magnetic structure contains only permanent magnets arranged in a modified Halbach array (Halbach, 1983 ▸), see Fig. 2 ▸, where an additional pair of magnets horizontally magnetized enhances the on-axis field. On the one hand, the absence of ferromagnetic poles reduces the non-linearity of the system (the field could be in good approximation regarded as the superposition of the individual pole contributions) and thus simplifies the operation, while on the other hand increases the stray fields and the forces between poles (especially along the beam axis) and reduces the maximum *K*-value. Each magnetic pole can be independently displaced vertically with a servo motor and precisely positioned by means of an absolute angular encoder, which allows for novel magnetic configuration discussed in §4. This flexibility requires high reliability of the individual components because of the large number of motors and encoders: if one pole does not move to the target position or worse becomes stuck, this can compromise the transport of the beam and the operation of the entire downstream beamline. Advanced control software has been developed as well to avoid the manual operation of the individual degree of freedom which can generate unnecessary beam losses. In the following subsections, the magnetic and mechanical designs of the two modulators are presented with particular care to their technical implementation but without the details of the simulation studies.

### 2.1. Magnetic design

**Figure 3:**
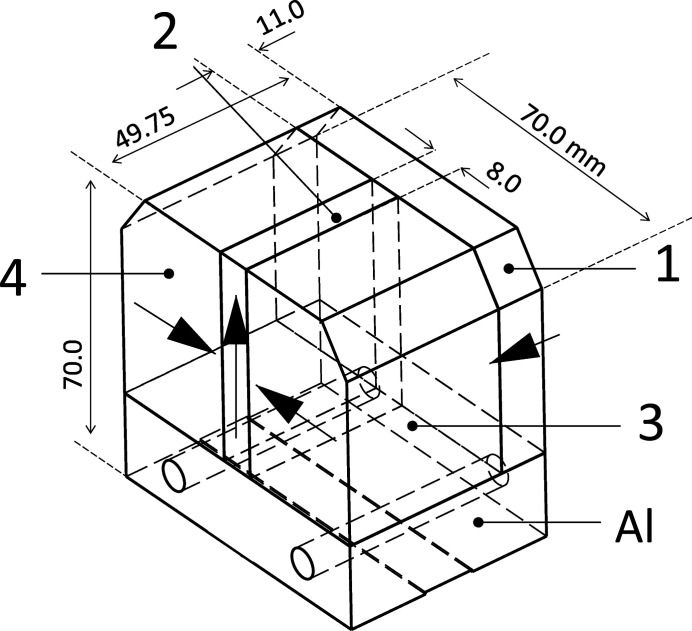
Detail of the production drawings of the north half-pole with the standard indexing and dimensions in millimetres. At the bottom is the aluminium (Al) support on top of which the four magnets are glued.

The building block of the magnetic structure is the half pole as presented in the top part of Fig. 2 ▸. It consists of four NdFeB (remanence, *B*_r_ = 1.2 T) magnets: one magnetized along the beam axis (1, *z*-axis), one vertically (2, *y*-axis) and two horizontally (3, 4, *x*-axis) with opposite directions. To build the modulator, this unit must be assembled in two distinct configurations, where each magnet of one assembly has the opposite magnetization direction than in the other one. In the bottom part of Fig. 2 ▸ the beginning of the modulator magnetic structure is presented using the two blocks and applying only a rotation around the *y*-axis. This results in a half pole followed by alternating full poles, for simplicity referred as 1/2, 1, 1 configuration. The choice of the half pole units was driven as well by the requested compatibility with the existing CHICs. They could be assembled in the future using these new components, thus reducing the field integral errors generated by the presence of the iron pole implemented in their present design. The magnetic optimization was performed using the software *RADIA* (Chubar *et al.*, 1998 ▸) and their final geometry is shown in the production drawings of Fig. 3 ▸. It is worth noticing that the maximum field amplitude is not achieved when magnets 1 and 2 have the same length along the *z*-axis, as is the case in a pure Halbach structure, because of the presence of the side magnets 3 and 4.

### 2.2. Mechanical design

**Figure 4:**
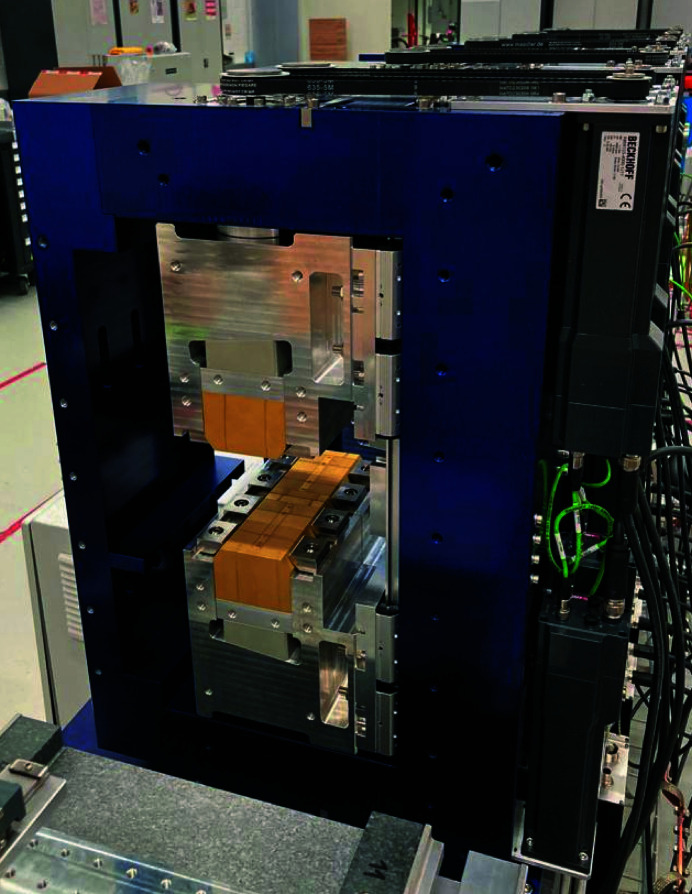
One magnetic period representing the mechanical unit of the modulator. In yellow are the magnets coated with TiN, on the right are the motors equipped with encoder and driving electronics, on the top is the gearbox made with a rubber chain, with a ratio of 1:3.

The poles are fixed on the modulator frame by means of mechanical clamps on the 45° short face of the magnets (Fig. 3 ▸). To minimize the forces required to keep them in place, the two half pole units are glued together to overtake the repulsive forces and consequently reducing the torque required on the fixing screws. Each period of the modulator is an individual mechanical unit with an independent closed frame equipped with two drive systems to change the distance between the upper and the lower poles (this distance is called the *gap*) from 11 to 90 mm. At the minimum gap a hard stop system is installed and carefully calibrated to avoid colliding with the vacuum chamber. This unit is presented in Fig. 4 ▸, where the four motors, the absolute rotary encoders and chain driven gearboxes (ratio 3) are visible. While assembling one of these units, the attractive force (mainly along the *z*-axis) between the positive and negative poles reaches about 1000 N and requires a reinforced guiding system with respect to the one originally planned for the CHIC (where the forces are lower and repulsive) to avoid a collision between the magnets. These forces vanish when several units are set together because of the periodic nature of the modulator: one pole is attracted both by its left and right neighbour resulting in zero net longitudinal force. Nevertheless, this is not the case at the extremes where this symmetry is broken, neither if one pole is accidentally or purposely (see §4.3) moved out of the magnetic axis. In both these cases, the system can be safely operated without risks of collision thanks to the double vertical guiding system implemented on each of the four poles. However, there are residual longitudinal displacements up to 0.2 mm when extreme configurations are reached (single pole fully out and the remaining fully in at minimum gap). During the magnetic optimization the closed frame of the individual units can be open from one side (preferentially at open gap, 90 mm) to access the magnets and applying, if required, mechanical shims on top and/or on the side of the magnets. This was used for tuning the vertical orbit when spurious horizontal field components (ideally all zero) were accumulating and giving rise to a net vertical displacement of the electron beam.

## 3. Magnetic measurements

The magnetic field was measured and calibrated with a Senis Hall probe (Popovic *et al.*, 2007 ▸), measuring the three components of the magnetic field on-axis. The first field integrals (Clarke, 2004 ▸) were measured with the moving wire to avoid systematic errors due to the unavoidable Hall probe calibration uncertainty (in particular between the positive and negative branches of the *V* ∝ *B* curves) and by the residual Hall planar effect, especially while measuring the *x*-component.

### 3.1. Alignment

**Figure 5:**
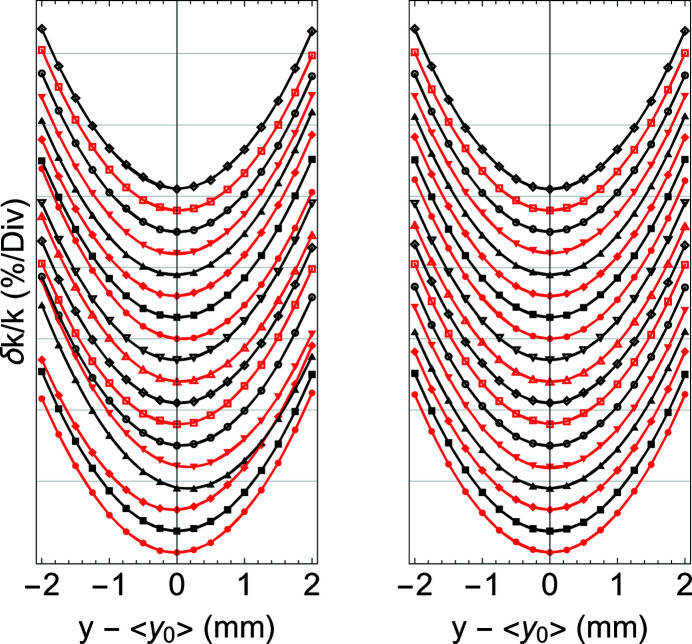
(Left) Relative variation of the local *K*, *k*_*n*_, for each pole, as a function of the vertical position before correction. (Right) The same quantity after corrections. On the vertical scale an offset is added between each pole to better distinguish their individual characteristics.

The magnetic field profile along the beam axis was measured at a fixed gap of 14 mm for different vertical (*y*) positions around the nominal beam axis height. To align all poles with respect to each other along a straight line, the parameter *k*_*n*_, hereafter called the local *K* (Pflüger *et al.*, 1999 ▸), is defined as the magnetic field integral between two nearest zeros, *z*_*n*_ (zeros of the magnetic field), as follows, 

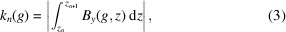

where for *k*_1_ and *k*_18_ the zeros value shall be chosen according to the extremes of the measurement region: *z*_1_ as the starting position and *z*_19_ as the end position. In Fig. 5 ▸ the relative variation, δ*k*_*n*_/*k*, as a function of the vertical position, before and after the alignment corrections are presented for all poles: the vertex of the parabola, *y*_0_, represents the centre of the magnetic field and it is used as the reference for the alignment.

### 3.2. Calibration

**Figure 6:**
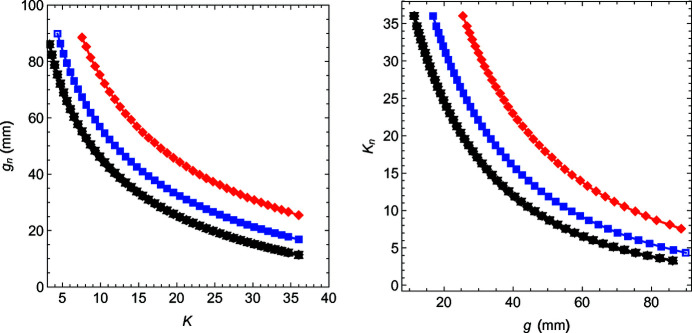
On the left in red: *g*_1_ and *g*_18_ (on the right: *K*_1_ and *K*_18_); in blue: *g*_2_ and *g*_17_ (*K*_2_ and *K*_17_); in black: *g*_3_ to *g*_16_ (*K*_3_ to *K*_16_). On the left side, the data are as-measured; on the right side, the data have been rearranged and fitted for the operation.

The magnetic profile of the HERO modulator consists of a half pole followed by full poles which yields a beam orbit wiggling off-axis at a distance that depends on the electron beam energy and field strength, *i.e.* the *K*-value. Having an even number of full poles, this configuration produces an output offset (no systematic net kick) of about the size of the wiggling amplitude, thus changing the input axis and leaking dispersion. For all those previous reasons, we decided to optimize the orbit on the alternative scheme consisting of 1/4, 3/4, 1 pole strength. This approach works as expected only for ideal magnetic poles, well spatially localized, but for real poles (especially those in the ends of the modulator which produce long tails) it shall be modified as following: 1/4+ε, 3/4+ε, 1. Estimating the value of ε is not trivial and it depends on the gap. A practical approach is to feedback its value on the results of the measurements as follows, 

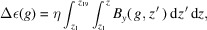

where Δε is the correction which requires a suitable choice of η.^2^ The first step of this optimization consists of measuring the profile for several gaps while all poles are set at the same gap. Secondly, estimating the local *K*-value, *k*_*n*_, as defined in equation (3). In the second measurement a new set of gaps shall be estimated using the new value of *k*_*n*_(*g*) following this algorithm: estimate *k*(*g*) as the average local *K* between *k*_3_ and *k*_15_; evaluate the new set of gaps solving the following set of equations, *k*_1_(*g*) = *k*/4, *k*_2_(*g*) = 3*k*/4, *k*_3_(*g*) = *k*,…, *k*_16_(*g*) = *k*, *k*_17_(*g*) = 3*k*/4, *k*_18_(*g*) = *k*/4. This procedure shall be iterated usually twice to obtain a stable result where only offsets (and not kicks) are left. Now, it is possible to refine these results using equation (3) and iterating with the following set of modified equations: *k*1(*g*) = (1/4 + ε)*k*, *k*_2_(*g*) = (3/4 + ε)*k*, *k*_3_(*g*) = *k*,…, *k*_16_(*g*) = *k*, *k*_17_(*g*) = (3/4 + ε)*k*, *k*_18_(*g*) = (1/4 + ε)*k*. Depending on the choice of the parameter η, this procedure could require more or less iterations. In Fig. 6 ▸(*a*), the results of this optimization are reported for the gap of the individual poles, *g*_*n*_, as a function of the actual (average) *K*-value. For compatibility with the literature and the standard naming at the Athos beamline the previous curves are inverted and fitted with an exponential function, 

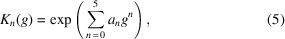

and the coefficients stored in the control system database for the operation.

## 4. Advanced operational modes

**Figure 7:**
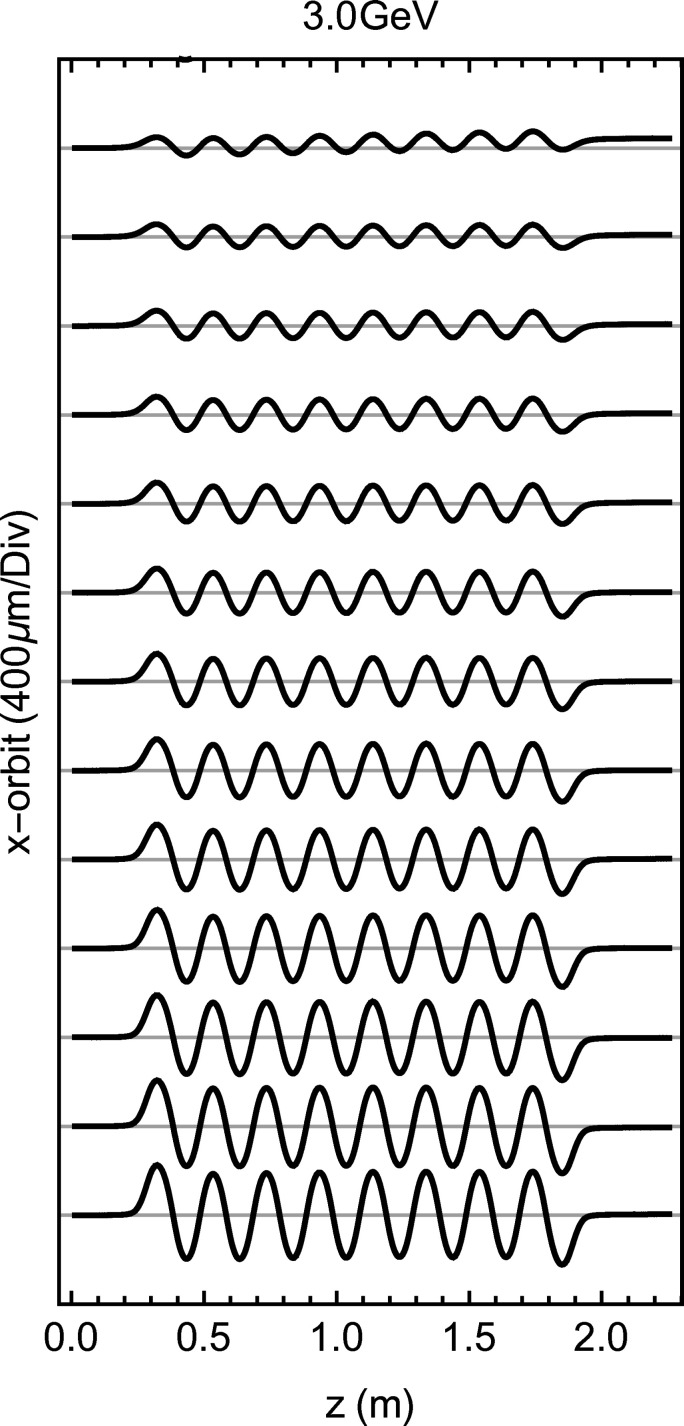
The orbit within the modulator for the operational *K* range between 8 (top) and 36 (bottom). The orbit is estimated for an electron beam energy of 3.0 GeV.

Using equation (5) it is possible to operate the modulator in its standard mode: it is sufficient to set a *K*-value and calculate back 18 gaps, *g*_*n*_. This allows an operational range of 8.0 < *K* < 36.0 corresponding to a wavelength range between 95.4 nm and 1875.0 nm for a 3.0 GeV electron beam. It is nevertheless possible to operate down to 15.9 nm (*K* = 3) with the penalty of an offset in the electron beam orbit at the end of the modulator, since first *g*_1_ and *g*_18_ and then *g*_2_ and *g*_17_ reach the 90 mm limit. In Fig. 7 ▸ the orbit for several *K* values is presented, starting from the bottom with the one calculated out of the strongest field amplitude using the formula 

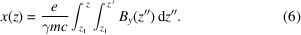

Residual upstream kicks are left over at very low *K* which generates offsets below 50 µm which could have been further minimized with more optimization iterations but they are nevertheless cancelled during the operation with beam by the orbit feedback.

### 4.1. Taper operation

To operate the modulator with a linear taper, it is sufficient to set the individual poles at their value as follows, 

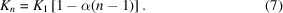

**Figure 8:**
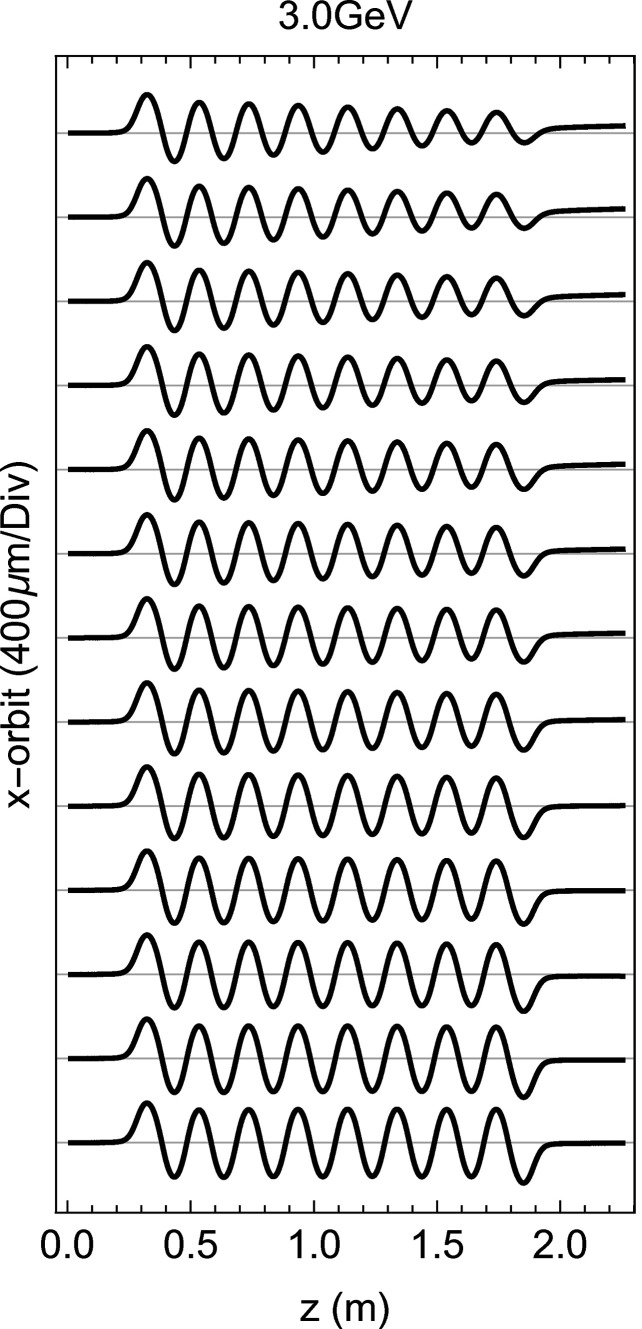
The orbit in the modulator around the maximum *K* value for different linear taper settings.

The magnetic measurements were performed for different taper values (α) and indicate that for a strong taper value there might be a small but non-negligible exit angle, see Fig. 8 ▸. This is generated around the entrance and can be corrected using the upstream correction steerers: we suggest for easy operation to use the orbit feedback which will naturally correct it. In this mode, the operation is limited by the minimum gap of *g*_1_ and the maximum gap of *g*_18_. For an optimum straight orbit, the taper shall be started from the very beginning (*K*_1_) up to the last pole. Reverse taper, if required, can be applied as well in a very similar way.

### 4.2. Triple period length

**Figure 9:**
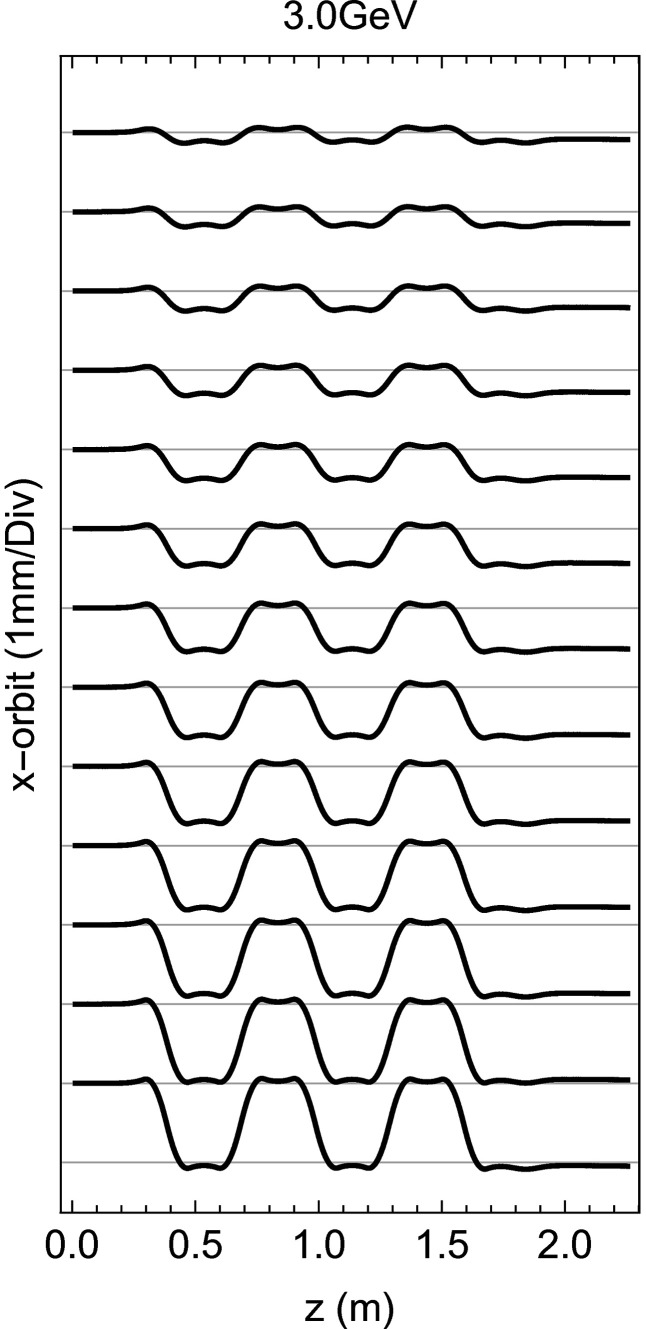
The orbit within the modulator for several gaps when operated in the triple period length mode. Note that the vertical division scale has been increased to 1 mm because of the larger amplitude of this configuration.

One additional mode identified as potentially useful for the operation of this modulator is the *triple period* with which much longer resonance wavelengths are possible, above 4 µm. It can be achieved keeping the poles (1, 4, 7, 10, 13, 16, 17, 18) at the maximum gap (90 mm, *off*) and the remaining ones closed at the strength required. This mode reduces the number of equivalent resonance poles to three and generates a beam horizontal offset with an unavoidable non-zero dispersion as clearly visible in Fig. 9 ▸. Both these parameters have to be carefully considered when operating the modulator in this exotic configuration.

### 4.3. Double *K* operation: *K*_*A*_ *K*_*B*_

**Figure 10:**
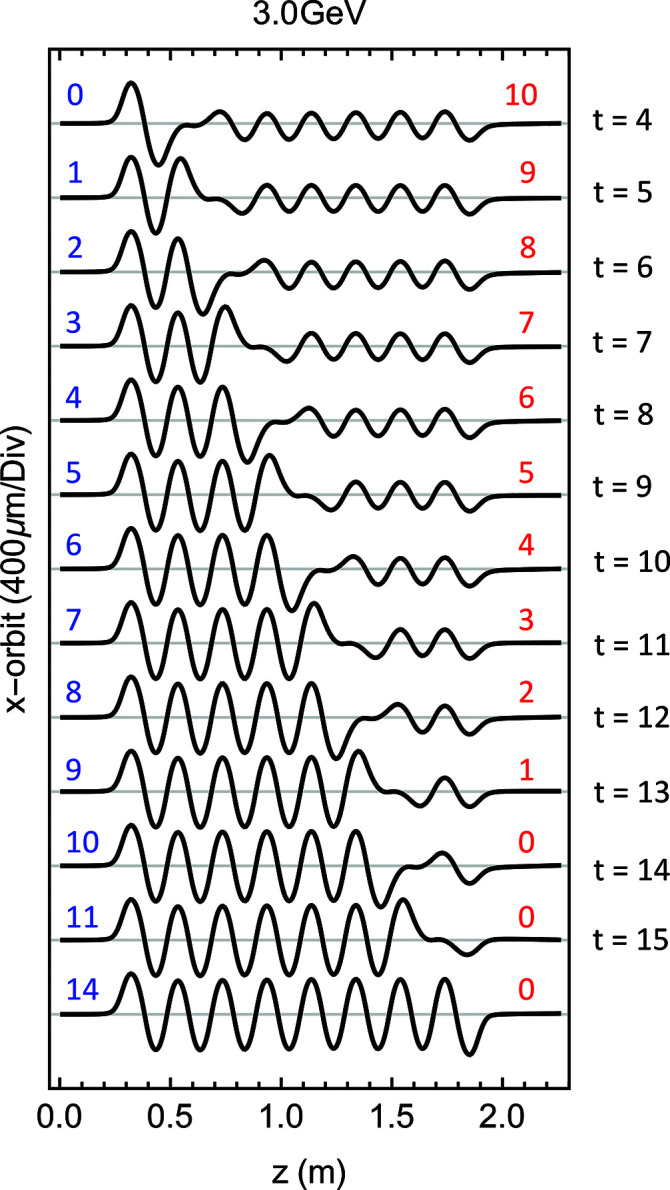
The orbit in the modulator for several possible arrangement of the *K*_*A*_ *K*_*B*_ configuration, starting from the bottom with only *K*_*A*_. In blue is the number of resonant poles at *K*_*A*_ and in red that at *K*_*B*_.

**Figure 11:**
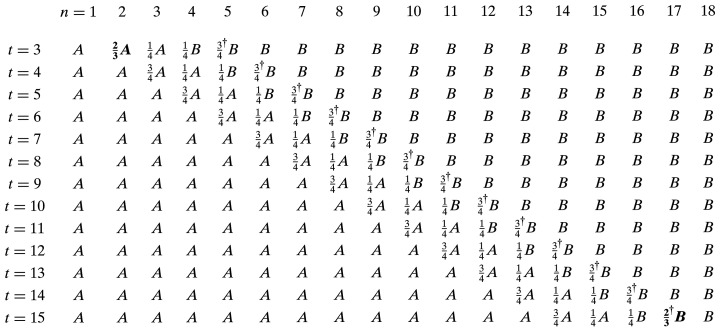
The full matrix (13 × 18) of the coefficients; for simplicity’s sake *K*_*A*_ and *K*_*B*_ are indicated with *A* and *B*, respectively – see text. To improve the small but non-negligible kick between the two modulators, at position *n* = *t* + 2 the coefficient (indicated with a † sign) has to be reduced by about a percentage: the measured value was saved in lookup tables for the operation.

The most advanced mode available and magnetically tested of this modulator consists of splitting the available poles into two families, with different *K*-values, *K*_*A*_ > *K*_*B*_. To keep a straight orbit, the *K*-values cannot be set with a sharp transition, but a smooth connection between the two sides needs to be organized as follows, 

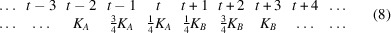

where *t* is the pole number where the first modulator finishes and *t* + 1 where the second starts. This mode was initially designed to detune pole by pole the modulator without losing the electron beam and for this purpose it was optimized with *K*_*B*_ set at its minimum possible value, 13.0. An example of this configuration is presented in Fig. 10 ▸, where the magnetically measured orbit is presented for a quasi-continuous transition (from bottom to top, 14 resonant poles, 11, 10, 9 down to zero) between the modulator *A* and *B*. Nevertheless, it is possible to set arbitrary values of both *K*_*A*_ and *K*_*B*_ but this approach was not extensively validated in the magnetic laboratory and will require extra care when commissioning it with beam. Before concluding, it is important to remark that equation (8) is valid for 3 < *t* < 15; for the extreme cases of *t* = 3 and *t* = 15, the value of 3/4*K*_*A*_ and 3/4*K*_*B*_, respectively, are not correct because there are only three poles left and the only sequence which gives both zero for the first and second field integral is 1/2, 1, 1/2. But since that specific pole is already a hidden 3/4, to transform it into a half the correct coefficient is 2/3 in front of *K*_*A*_ when *t* = 3 and for *K*_*B*_ when *t* = 15. Here, for the sake of clarity the full matrix (13 × 18) of the coefficients is shown in Fig. 11 ▸ using the nominal value of the coefficients, which already give reasonably straight orbits. To improve the small but non-negligible kick between the two modulators, at position *n* = *t* + 2 the coefficient (indicated with a † sign) has to be reduced by about a percentage: the measured value was saved in lookup tables for the operation.

## 5. Conclusions and outlooks

**Figure 12:**
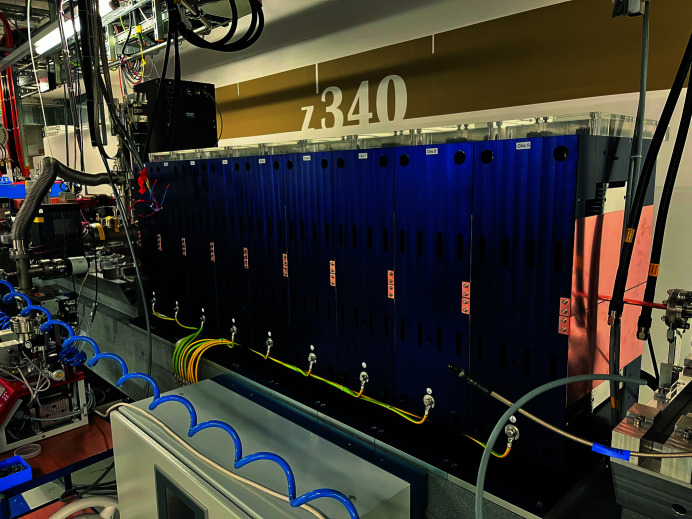
The second modulator downstream in the SwissFEL tunnel. This unit was already tested with an electron beam of about 3 GeV.

The two HERO modulators have been designed, assembled, magnetically tested and installed in the SwissFEL tunnel (Fig. 12 ▸). The large degrees of freedom, made possible by the compact and modular design, have been used to implement advanced operational modes, such as the *K*_*A*_ *K*_*B*_ mode which to our knowledge has never been investigated in previous projects. The second modulator (the one most downstream) has been very recently tested as well with beam at the SwissFEL where it demonstrated a net energy transfer from an external laser tuned at 800 nm. The full setup will be tested with beam in 2023. A preliminary assessment of micro-bunching instability excludes that the residual field of the modulator can spoil the beam for normal SASE operation; it would require a significantly longer beamline to accumulate an energy modulation from the micro-bunching. On the contrary, wavelengths longer than 100 nm should be excited to not produce coherent emission and self-modulation from the current spike of the Athos beam tail, thus a *K* value lower than 8.6 (at 3.15 GeV, the new reference electron beam energy of Athos) shall be set when the modulator is not in operation.

The magnetic assessment of these two prototypes is very satisfactory but the system could be further improved by changing the design of the poles to compensate magnetically the longitudinal forces. This shall reduce from one side the requirement on the vertical guiding system and improve the position accuracy of the poles especially along the beam direction. This upgrade could be done as well to compensate the vertical forces even if they were not at all a concern. If required, stronger fields are achievable by substituting the vertical magnet (the one magnetized along the *y*-axis) by a CoFe pole. Differently from the CHIC configuration, where the central pole splits to introduce horizontal offsets, in the modulator configuration the non-linearity introduced by the iron pole can be harnessed to produce no net field integral (spurious kick).

## Footnotes

1 From the design and procurement phase of the two modulators, the extraction energy from the main linac was increased from 3.0 GeV to 3.15 GeV, shifting the tuning range from 2.9 to 3.4 GeV. With these new parameters a higher maximum *K* of 38 should have been specified to cover all possible options.

2 η was evaluated experimentally measuring the double integral IIB for two closed values of ε with the following formula: η = (ε_2_ − ε_1_)/(IIB_2_ − IIB_1_).
